# A methodology for evaluating salespeople performance considering efficiency and effect: A case study of a liquor company in China

**DOI:** 10.3389/fpsyg.2022.923198

**Published:** 2022-08-12

**Authors:** Aixin Cai, Maohong Liu, Huan Liu

**Affiliations:** ^1^School of Economics and Management, Wuhan University, Wuhan, China; ^2^School of Evergrande Management, Wuhan University of Science and Technology, Wuhan, China; ^3^Research Center for Marketing Engineering and Innovation, Wuhan University, Wuhan, China; ^4^Disaster Prevention Research Institute, Kyoto University, Kyoto, Japan

**Keywords:** data envelopment analysis, Gini impurity, performance evaluation, decision tree, salespeople management

## Abstract

The performance evaluation is one of the most important organizational management strategies used to guide the sales behavior of sales staff. However, it should be work process-oriented or sales result-oriented has become a dilemma for the management when evaluating employees' performance. Therefore, comprehensively evaluating the work process and sales results has become a challenge when it comes to salespeople performance appraisal. To solve this dilemma, this research builds a salesperson performance evaluation model which considering both of work efficiency and effectiveness, based on the decision tree model. Specifically, we use the Data Envelopment Analysis (DEA) model to quantify the work efficiency of salespeople and measure the work effectiveness by amount of sales. Moreover, this research proposes an advanced integrated DEA model by integrating the self-evaluation DEA models, peer-evaluation DEA models, and Gini impurity, which is identified to be more stable compared with the current DEA model. Finally, a case study of a Chinese liquor company is introduced to illustrate the applicability and feasibility of the salesperson performance evaluation model. The proposed model is applied to evaluate the performance of the salespeople, and a set of comprehensive and objective sales performance evaluation results are obtained. The estimated results can provide feasible sales management suggestions for the company in diagnosing work problems of salespeople.

## Introduction

The sales force is one of the most important cogs for companies, because salespeople play a fundamental role in marketing strategy implemental (Kumar et al., 2014). Their ability and performance are directly related to enterprises' interests and competitiveness in the market (Han and Fan, 2021). Therefore, it is crucial to evaluate their achievements scientifically to improve corporate performance (Grafton et al., 2010). However, companies are faced with a dilemma when conducting performance appraisals on salespeople: should the appraisal be result-oriented or process-oriented? If it is result-oriented, the objective data can guarantee fairness and transparency. But it might overlook the efforts paid by the sales staff and market environment differences (Mackenzie et al., 1993; Panagopoulos et al., 2017). If the appraisal is process-oriented, it might make the conversion relationship between work input and output unclear, though it considers the behavioral process. To be more specific, it is highly subjective, which might not align with the evaluating standards and cause the “halo effect” (Mallin et al., 2022). So, a method of salesperson performance measurement that can effectively connect the personal input and work output of the salesperson, and incorporate both objective and subjective measures is very necessary (Churchill et al., 1985). However, in sales research, this integrated approach is rare, with most studies using either result-oriented or process-oriented measurement (Bolander et al., 2021).

According to one of the most widely held definitions, salesperson performance is the salesperson's contribution to the goals of organization (Churchill et al., 1985). This contribution can take the form of sales productivity and behaviors that are the precursors to sales productivity (Hall et al., 2015). Productivity refers to the contribution of salespeople to organizational performance, which is synonymous with effectiveness (Ahearne and Lam, 2012). Campbell et al. (1993) suggest that since the complex behaviors that salespeople enact tend to vary across selling environment, performance is better thought of as inputs and outputs of effort quantity and quality (e.g., strategy) of a salesperson. As we allude to subsequently, a performance evaluation is a management concept reflecting the efficiency and effectiveness of input and output. From these perspectives, our working definition of salespeople performance is the salespeople's contribution to the organization's goals and to the effective functioning of the organization.

After reviewing existing studies on the performance measurement and management literature, the authors found that many scholars have combined input and output from the perspective of efficiency, and measured performance by relative efficiency (Cook and Hababou, 2001; Cooper, 2005). Efficiency refers to the ratio of sales outcomes and inputs. It shows the quality of a salesperson's effort by comparing inputs (e.g., calls, customer visits) to outcomes (e.g., calls, customer visits) (Campbell et al., 1993). Efficiency is central to salespeople performance evaluation that relies on DEA method (Charnes et al., 1978). Since Charnes et al. formally proposed the first DEA model-the CCR model, a series of important DEA models from different perspectives have been proposed, such as the BCC model proposed by Banker et al. (1984), the additive DEA model proposed by Charnes et al. (1982) and the super efficiency model proposed by Andersen and Petersen (1993). Also there exist different viewpoints for utilizing the DEA technique, for instance, input-oriented and output-oriented views (Cooper et al., 2007). These DEA models have been widely used in performance evaluation. For example, Boles et al. (1995) proposed the use of DEA to evaluate salespeople. Yeung and Azevedo (2011) analyzed 27 state courts' management performance in Brazil with relative efficiency. And they found that court management performance is related to court efficiency. From the perspective of internal bank operations, Chu et al. (2021) constructed a two-stage network DEA model in line with the bank's structural characteristics, and measured the efficiency of 23 commercial banks. Cook and Hababou (2001) extended the additive DEA model to evaluate sales performance within a major Canadian Bank. However, both of these studies are based on only one DEA model individually to assess the performance. It is clear that the results derived from various DEA models may not be the same (Chen, 2014). Each of the above-mentioned models have some valuable advantages which we would like not to ignore. But they have some disadvantages, too (Soleimani-Damaneh and Zarepisheh, 2009). For instance, if a decision-making unit (DMU) has a strict minimum input value for any input item, then it is recognized as an efficient unit when being assessed by some DEA models, such as BCC model without any weight restrictions, even if it is very poor with respect to other factors (Cooper et al., 2007). This alarms us about using the results of one DEA model individually. Hence, any evaluation from only one perspective is considered as one-sided (Wang et al., 2007). In regard to this, it was recommended that different DEA models be integrated according to certain scientific rules for an overall performance evaluation, which can compensate for these pitfalls (Wang and Luo, 2006). For example, Soleimani-Damaneh and Zarepisheh (2009) employed Shannon's entropy to integrate a family of efficiency scores of various different DEA models, in an attempt to give a complete performance ranking. However, this method is relatively aggressive as it may lead to problems such as too large a difference in the weights assigned to the results of each DEA model.

In addition, the DEA evaluation method overemphasizes the conversion efficiency of input and output. But high work efficiency does not promise high work output. In other words, relative efficiency cannot effectively reflect work results. Therefore, evaluating performance based on efficiency is not always effective. In order to build a performance evaluation model that comprehensively considers sales results and behavioral processes, the above two problems must be solved. Namely:

- How to balance the emphases of different DEA models and maximize their advantages to effectively measure the comprehensive work efficiency?
- Then, on this basis, how to effectively visualize the work output and solve the problem of balancing the behavioral process and results in the salesperson performance evaluation system?

Although previous studies on organizational performance evaluation mostly used relative efficiency to measure performance (Aydin and Yurdakul, 2021), a performance evaluation is different from an efficiency evaluation (Kerr and Marcos-Cuevas, 2022). In evaluating salespeople performance, it is important to distinguish between efficiency evaluating and performance evaluating. The former is from the engineering perspective that its evaluation index emphasizes the measurement of the input-output ratio of decision-making units (Ruiz and Sirvent, 2019). It shows the quality of a salesperson's behaviors (Campbell et al., 1993). Conversely, performance evaluation is a management concept with multiple meanings. According to the definition of salespeople performance, it requires to reflect sales productivity and behaviors (Hall et al., 2015). Productivity is synonymous with effectiveness, which can be outcomes (e.g., sales revenue) (Ahearne and Lam, 2012). Thus, in evaluating salespeople performance, we should take into account the efficiency as well as the effectiveness of input-output activities (Krapfl, 1982; Hunter and Perreault, 2007; Katsikeas et al., 2018; Gilbert, 2019). The effectiveness can be measured in terms of sales (Ahearne and Lam, 2012), which reflects the sales results. Generally, the higher the total operating income, the better the effect of the output, and vice versa (Chen, 2010). Therefore, a salesperson performance evaluation is a systematic evaluation index that effectively combines sales results and behavioral process by considering both work efficiency and work effectiveness.

With the development of the mobile internet, the work environment of sales staff is gradually digitized and scene-based. And such development has made relevant data on the work process of sales staff, such as working hours, trajectories, the number of customers visited and other specific data viable, which provides a sound data basis for building a salesperson performance evaluation system that comprehensively reflects behavioral process and results. In order to solve the first problem, this paper resorts to the Gini classification criterion from the CART decision tree model (Lei and Qiu, 2021), which is for measuring the order degree of the set, to examine the discriminative power of the work efficiency results obtained from various DEA models (Luna et al., 2019). Furthermore, we proposed an integrated DEA efficiency evaluation model based on self-evaluation (Boyd and Fare, 1984; Kleine, 2004) and peer-evaluation DEA models (Doyle, 1994; Liu, 2018) and Gini impurity weighting (Soleimani-Damaneh and Zarepisheh, 2009). In addition, we have incorporated indicators such as working hours, customer visits, and regional market potential into the DEA model to improve the reliability of the results. As for the second problem, we tend to measure the behavioral process by evaluating the work efficiency of the sales staff and measuring the work results by the sales. Inspired by the decision tree model, we constructed a systematic performance evaluation system that could effectively combine the sales results and the behavioral process.

To sum up, due to the difficulty in obtaining the work process data and the limitations of the methods, most of the existing salespeople performance evaluation researches focus on theoretical analysis and subjective measurement (Wiemann et al., 2019; Kerr and Marcos-Cuevas, 2022). While a small amount of objective measures are focus on relative evaluation (e.g., efficiency) (Ahearne and Lam, 2012). An effective connection and evaluation between the salesperson's personal input and work results considering their sales performance is still lacked and needed. To fill this gap, this research has constructed a performance evaluation model that effectively reflects the work process and work results. Taking the sales data of a liquor brand in China for a case study, we tested the validity of the model, analyzed the performance evaluation results, and put forward targeted management suggestions. Moreover, this performance evaluation method could provide new ideas and solutions for commodity sales companies to identify potential problems and improve sales staff performance. The main contributions of this research are as follows:

- In this study, the concept of work efficiency and effect are taken into consideration while evaluating sales performance of salespeople, and a salesperson performance evaluation model that comprehensively considers the work process and work results is established.
- A Gini impurity-based DEA model that can effectively integrate self-evaluation DEA models and peer-evaluation DEA models is proposed, which is more stable compare with the current DEA model.
- A case study is presented.

The reminder of this research is organized as follows: In the next section, the advanced DEA model is proposed and illustrated. In Section Data and variables, the variables and data of the empirical model are briefly presented. In Section Results, the salespeople performance evaluation model is presented, and the results are given. Finally, discussion and conclusions are presented in Section Discussion and conclusion.

## The work efficiency measurement model

### Basic DEA model

Consider *N* DMUs, which use *t* inputs (*i* = 1,2,…, *t*) to produce *s* outputs (*r* = 1,2,…, *s*). Let *DMU*_*k*_ (*k* ϵ {1,2...,*N*}) to be the kth DMU under evaluation. For *DMU*_*k*_, let the ith input as *x*_*ik*_ and the rth output as *y*_*rk*_. Then the efficiency value of *DMU*_*d*_ measured by the CCR model, denoted as $E_{dd}^{I-CCR}$, is calculated as follow:


(1) $$\{\begin{array}{c} \;\max E_{dd}=\sum_{r=1}^{s}u_{rd}y_{rd}\\ s.t.\\ \sum_{r=1}^{s}u_{rd}y_{rd}-\sum_{i=1}^{t}v_{id}x_{id}\leq 0\\ u_{rd},v_{id}\geq 0\\ \;r=1,2,\ldots ,s;\;i=1,\ldots ,t \end{array}$$

In the formula, the variables *v* and *u* are the weights given to the ith input and the rth output when evaluating *DMU*_*d*_. Based on the CCR model, researchers have developed a variety of basic DEA models (such as the BCC model) to accord with various problems. However, these traditional DEA models take a self-evaluation perspective, leading to the case that many DMUs are evaluated as efficient and cannot be discriminated any further. In order to resolve the self-evaluation defect of the traditional DEA method, Sexton et al. (2010) proposed a cross-efficiency evaluation method with self- and peer-evaluation. However, the new model also has its own defects.

Since different DEA models measure efficiency scores from different perspectives, the rankings of DMUs obtained by these different DEA models may not be the same (Chen, 2014). Some questions arise: it may be not easy for one to decide which model is more suitable, and which model's result is more desirable. As noted in Soleimani-Damaneh and Zarepisheh (2009) one may need to try several different models to measure the efficiency scores of DMUs. Besides, each DEA model has some precious advantages that we would like not to ignore (Lee, 2019). It is also worth noting that if we cannot identify the production frontier by preliminary survey, it may be risky to rely on only one DEA model (Cooper et al., 2007). Hence, it is wise to combine the results of the different models. To this end, we draw on the idea of Soleimani-Damaneh and Zarepisheh (2009) to provide an approach and give a complete ranking. Specifically, we will use Gini impurity to judge the certainty of various DEA models, and weight them according to the degree of certainty. Finally, an integrated DEA model will be constructed to evaluate the relative efficiency of the DMUs to calculate the efficiency sequence more consistent with the actual situation.

### Gini impurity

#### Definition

Gini impurity $gini\left(T\right)=\overset{j=m}{\underset{j=1}{\sum }}p_{j}\left(1-p_{j}\right)$ (j = 1,2,…,m), where$p_{j}=\frac{f_{j}}{N}$ (*N* is the sum total of the samples in the decision-making unit set *T*), *p*_*j*_represents the probability of the occurrence of the *j*-type decision-making unit in a certain DEA model, 0 ≤ *p*_*j*_ ≤ 1; *f*_*j*_ is the frequency of Category *j*, and *m* is the number of decision-making unit categories, *m* ≤ *N*. Gini impurity gini(*T*) refers to the probability of two randomly drawn samples from Dataset *T* whose class labels are inconsistent.

#### Properties

The more concentrated the efficiency value of the decision unit, the smaller the gini(*T*), and the greater the ambiguity of the corresponding DEA model results. On the contrary, the greater the difference between the efficiency values of the decision unit, the greater the gini(*T*), and the greater the certainty of the corresponding DEA model results. When all DMU efficiency values are the same, gini(*T*) reaches the minimum value zero, and the DEA model is the least determinate.

The properties above can be proved by mathematical methods. Let *M*_*i*_ be the efficiency value of a DMU in a certain DEA model. For ∀ *i,k* ϵ [1,2,…, N], *M*_*i*_ = *M*_*k*_, that is, the efficiency value of all DMUs is the same, known from $p_{j}=\frac{f_{j}}{N}$, at this time $p_{j}=\frac{N}{N}=1,\;j=1$. So in this case, Gini impurity $G_{1}=gini\left(T\right)=\overset{j=m}{\underset{j=1}{\sum }}p_{j}$ (1−*p*_*j*_) = 0.

Assume that in a certain type of DEA models, ∃ *i,k*ϵ[1,2,…, *N*], when *i*≠*k*, *M*_*i*_≠*M*_*k*_, that is, the efficiency values of some DMUs are different, then $p_{j}=\frac{1}{N}+\varepsilon_{j}$, ε_*j*_reflects the degree of dispersion of the DMU efficiency value, 0 < ε_*j*_ <1. Then, the Gini impurity is:


(2) $$\begin{array}{l} G_{2}=gini\left(T\right)=\Sigma_{j=1}^{j=m}p_{j}\left(1-p_{j}\right)=1-\Sigma_{j=1}^{j=m}{\left(p_{j}\right)}^{2}\\ =1-\Sigma_{j=1}^{j=m}{\left(\frac{1}{N}+\varepsilon_{j}\right)}^{2}\\ =1-\left(\frac{m}{N^{2}}+\frac{2}{N}\times \Sigma_{j=1}^{j=m}\varepsilon_{j}+\Sigma_{j=1}^{j=m}{\varepsilon_{j}}^{2}\right) \end{array}$$

Because $\overset{j=m}{\underset{j=1}{\sum }}p_{j}=\;1$,

So $(\frac{1}{N}+\varepsilon_{1})+(\frac{1}{N}+\varepsilon_{2})+\ldots +(\frac{1}{N}+\varepsilon_{m})=\;1$,


(3) $$\begin{array}{r} \text{That is }\varepsilon_{1}+\varepsilon_{2}+\ldots +\varepsilon_{m}=1-\frac{m}{N} \end{array}$$

Substitute Equation (3) into Equation (2):


(4) $$\begin{array}{l} G_{2}=1-\left[\frac{m}{N^{2}}+\frac{2}{N}\times \left(1-\frac{m}{N}\right)+\left(\varepsilon_{1}^{2}+\varepsilon_{2}^{2}+\ldots +\varepsilon_{m}^{2}\right)\right]\\ =1-\frac{2}{N}+\frac{m}{N^{2}}-\left(\varepsilon_{1}^{2}+\varepsilon_{2}^{2}+\ldots +\varepsilon_{m}^{2}\right) \end{array}$$

From the assumptions, we know that 2 ≤ *m* ≤ *N*, 0 < ε_*j*_ <1,

so:


(5) $$\begin{array}{r} 0{\leq \varepsilon }_{1}^{2}+\varepsilon_{2}^{2}+\ldots +\varepsilon_{m}^{2}\leq 1-\frac{4}{N}+\frac{4}{N^{2}} \end{array}$$

Substitute Equation (5) into Equation (4):


(6) $$\begin{array}{l} G_{1}<\frac{2}{n}+\frac{2}{n^{2}}\leq G_{2}=1-\frac{2}{N}+\frac{m}{N^{2}}\\ -\left(\varepsilon_{1}^{2}+\varepsilon_{2}^{2}+\ldots +\varepsilon_{m}^{2}\right)\leq 1-\frac{1}{n} \end{array}$$

Therefore, according to the right-hand side expression of Equation (6), when the efficiency value of DMUs in a DEA model fluctuates greatly, the value of Gini impurity G_2_ will become greater, the differentiation degree of the sets will become higher, the certainty of the node will become greater. On the contrary, the value of Gini impurity G_2_ will become smaller, the differentiation degree of the sets will become lower, the certainty of the node will become smaller. When the efficiency values of all DMUs are the same, based on the left-hand side expression of Equation (6), the Gini impurity will reach the minimum value G_1_ = 0. That is, the discrimination degree of the efficiency value of DMUs will be the smallest, and the certainty of the node will become the smallest, too.

From a practical perspective, when the DMUs in a DEA model are all valid, they cannot be further sorted because the efficiency values of the valid DMUs are all 1, resulting in the low certainty of the evaluation results of the model and a small Gini impurity. When DMUs can be clearly distinguished in a DEA model, the evaluation results of the model become more certain and Gini impurity is greater.

### The integrated efficiency model incorporating multiple DEA models and Gini impurity

After introducing and analyzing the concept of Gini impurity, we have proposed an algorithm for an integrated DEA model based on Gini impurity weighting. We used Gini impurity to evaluate the certainty of various DEA models, and assigned weights to each DEA model according to the degree of certainty. Finally, we have established an integrated DEA model to evaluate the relative efficiency of decision-making units. Details are as follows:

Step 1. Calculate the efficiency matrix *E*. Assume the set of efficiency results of DMUs in K DEA models is ∪ = {*M*_1_, *M*_2_, …, *M*_*K*_}, *T*_*l*_ represents the measured efficiency result of the *l*th DEA model. The following result matrix can be obtained:


$$\begin{array}{l} \;M_{1}\;M_{2}\;\ldots \;M_{K}\\ \;↓\;↓\;↓\\ E=\left[\begin{array}{ccc} \begin{array}{c} E_{11}\\ E_{21}\\ E_{31} \end{array} & \;\begin{array}{c} E_{12}\\ E_{22}\\ E_{33} \end{array}\;\begin{array}{c} \cdots \\ \cdots \\ \cdots \end{array} & \begin{array}{c} E_{1K}\\ E_{2K}\\ E_{3K} \end{array}\\ \vdots & \;\vdots \;\vdots \; & \vdots \\ E_{n1} & {\;E}_{n2}\;\cdots & E_{nK} \end{array}\right] \end{array}$$

Where *E*_*il*_ represents the efficiency value of *DMU*_*i*_ in the *l*th DEA model.

Step 2. Calculate the weights of various DEA models. Calculate the Gini impurity of a vector *M*_*l*_(*l* = 1, 2, …, *K*) by the definition $gini(T_{l})=\sum_{j=1}^{j=m}p_{jl}\left(1-p_{jl}\right)=1-\sum_{j=1}^{j=m}\frac{{\left(f_{jl}\right)}^{2}}{n^{2}}$. In order to ensure that the weight *w*_*l*_ of the *l*-th DEA model is in the range of 0–1 and $\overset{l=K}{\underset{l=1}{\sum }}w_{l}=1$, we normalized the Gini impurity gini(*T*) of various DEA models to obtain the weight *w*_*l*_ of each DEA model.

Step 3. Calculate the efficiency E of each DMU. Through the integrated DEA model based on Gini impurity weighting, we can obtain the objective, stable and unique efficiency value $E_{i}=\overset{l=K}{\underset{l=1}{\sum }}w_{l}\times M_{il}$ of *DMU*_*i*_(*i* = 1, 2, …, *N*).

### Salespeople performance evaluation model

From the work efficiency evaluation methods mentioned above, it could be said that work efficiency emphasizes the conversion efficiency between input and output. And high work efficiency does not always bring high output. It is one-sided to evaluate a salesperson's performance by work efficiency. According to the definition of salespeople performance, in evaluating salespeople performance, we should take into account work efficiency as well as work effectiveness, which can be outcomes (Hunter and Perreault, 2007; Ahearne and Lam, 2012; Gilbert, 2019). Relying on work input and sales output data to solve the problem of balancing the work efficiency and work effectiveness in salesperson performance evaluation, is a challenge (Bolander et al., 2021). Yeo and Grant (2019) proposed this challenge can be overcome by using decision tree analysis, given their data-centric nature, allowing the data to tell the story. As noted in Yeo and Grant (2019), decision tree identifies sales performance trends by drawing insights from various statistical relationships between different variables, and uses advanced analytics to identify the most valuable sales opportunities for sales performance. The results can be used to develop polices and strategies that radically improve their performance (Kim et al., 2011; Jeiad et al., 2018). For this reason, in this study we introduce a decision tree model to combine work efficiency and work effectiveness, and balance the behavioral process and results in the salespeople performance evaluation system.

**Figure 1:**
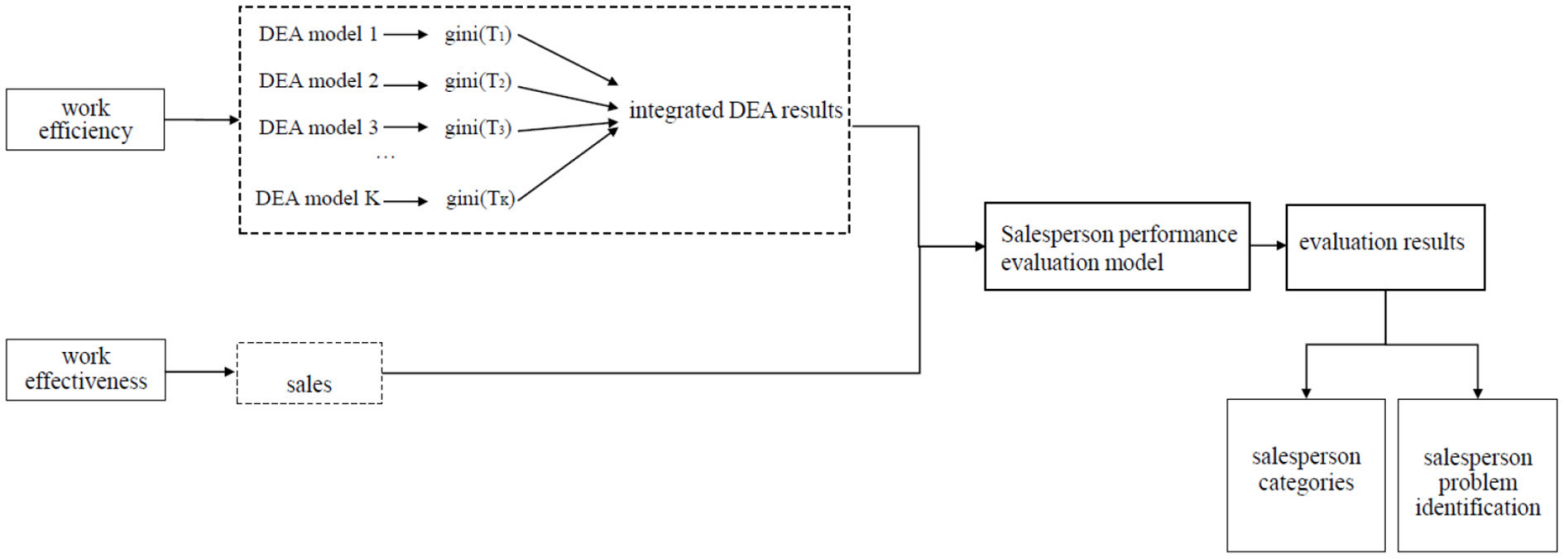
Salesperson performance evaluation framework and logic.

We have applied the decision tree idea to the salesperson performance evaluation. Drawing on Fumero and Vercellis (2001) and Soleimani-Damaneh and Zarepisheh (2009) ideas on using efficiency to measure performance, we have established a performance evaluation model for the salesperson that involves both process performance and sales results evaluations. Figure 1 illustrates the framework and logic to evaluate salesperson performance. Specifically, the sales results are measured by sales, and the integrated DEA model with Gini impurity is used to measure the work efficiency of the sales staff through indicators such as working hours and customer visits. The model has taken both the work process and work effect into consideration, thus making the performance evaluation scientific, objective and convincing.

**Figure 2:**
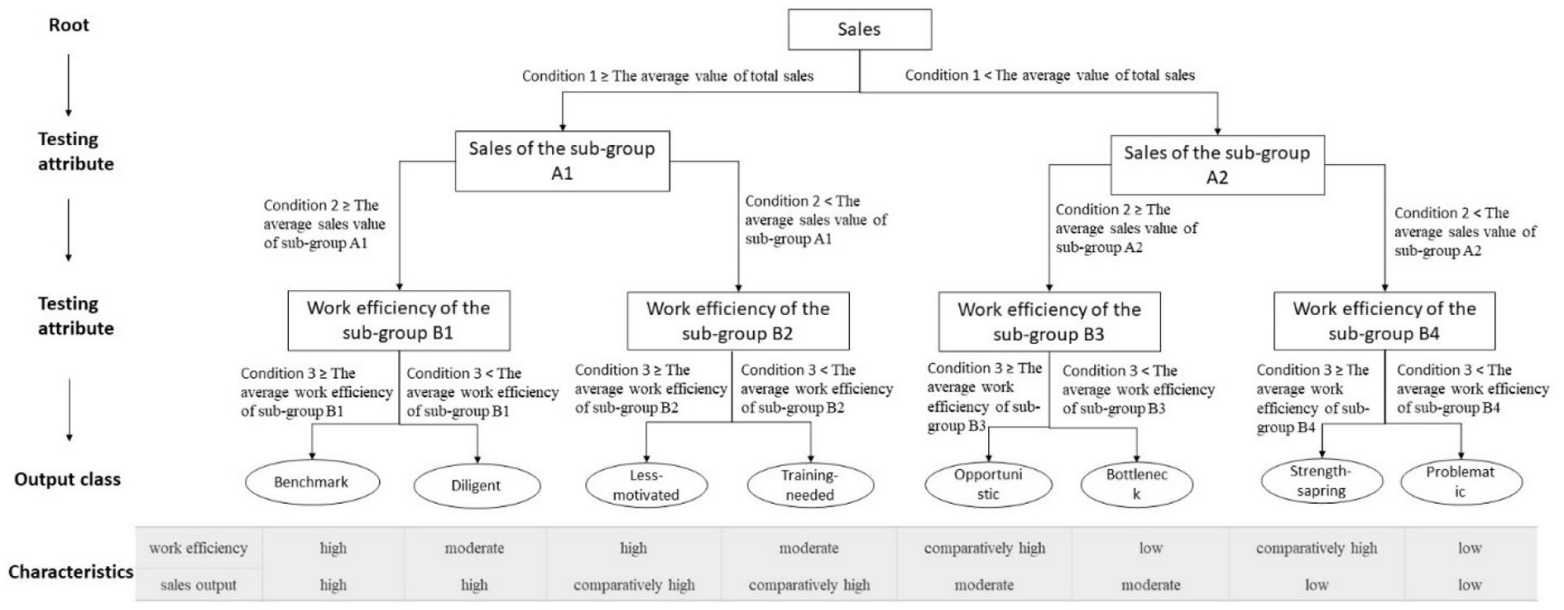
The salespeople performance evaluation model.

The work performance of the sales staff is evaluated and classified according to the performance evaluation model. Figure 2 illustrates the salespeople performance evaluation model. The salesperson mainly has two testing attributes, namely sales and work efficiency. Each internal node represents a testing attribute, and the leaf nodes represent the types of sales personnel. Firstly, the tree splits the salespeople into two groups, at the root nodes. Each group represents a decision node based on sales. Sub-group A1 includes records that satisfy Condition 1 ≥ the average value of total sales. Sub-group A2 includes records that satisfy Condition 1 < the average value of total sales. Secondly, both sub-group A1 and sub-group A2 continue to be split into two child nodes: sub-group B1, sub group B2 and sub-group B3, sub group B4. Sub-group B1 includes records that satisfy Condition 2 ≥ the average sales value of sub-group A1. Sub-group B2 includes records that satisfy Condition 2 < the average sales value of sub-group A1. Sub-group B3 includes records that satisfy Condition 2 ≥ the average sales value of sub-group A2. Sub-group B4 includes records that satisfy Condition 2 < the average sales value of sub-group A2. Finally, sub-group B1, sub-group B2, sub-group B3, sub-group B4 continue to be split into two child nodes based on efficiency, where records satisfying the splitting conditions exhibit the outcome represented by the terminal nodes. Specifically, eight categories can be made: benchmark, diligent, less-motivated, training-needed, opportunistic, bottleneck, strength-sparing and problematic salespeople. The terminal nodes, benchmark, includes records that satisfy Condition 3 ≥ the average work efficiency of sub-group B1. The terminal nodes, diligent, includes records that satisfy Condition 3 < the average work efficiency of sub-group B1. The terminal nodes, less-motivated, includes records that satisfy Condition 3 ≥ the average work efficiency of sub-group B2. The terminal nodes, training-needed, includes records that satisfy Condition 3 < the average work efficiency of sub-group B2. The terminal nodes, opportunistic, includes records that satisfy Condition 3 ≥ the average work efficiency of sub-group B3. The terminal nodes, bottleneck, includes records that satisfy Condition 3 < the average work efficiency of sub-group B3. The terminal nodes, strength-sparing, includes records that satisfy Condition 3 ≥ the average work efficiency of sub-group B4. The terminal nodes, problematic, includes records that satisfy Condition 3 < the average work efficiency of sub-group B4. The characteristics for each type of salesperson are summarized in Figure 2.

## Data and variables

### Data descriptions

The current research is a case study on a liquor enterprise in Wuhan City, Hubei Province. Its sales areas include Caidian District, Dongxihu District, Hankou District, etc. The product sales targets include standard food supermarkets, restaurants, liquor retail terminals, liquor wholesale supermarkets, comprehensive supermarkets and terminal direct-operated stores, etc. It should be noted that no regional competition exists between the sales staff responsible for the same product.

This research obtains data from the backend of the daily sales work data recording system of the liquor enterprise from June 15, 2018 to July 14, 2018. After informing the sales staff of the purpose of this research, we opened the daily sales work data recording system to 116 sales staff to help us understand the daily sales details. The system automatically obtains customer order information, obtains salesperson work input, and forms information on the daily sales work trajectory of salespeople. For example, when salesperson visits a customer, the system can automatically obtain the visit time and positioning. We extracted the basic data reflecting the work process of the sales staff according to the sales work data record. The basic data contains the following fields: salesperson's name, order creation time, product name, product unit price, product quantity, customer visit location and time, etc. Since some of the salespeople quit using the sales system in the middle of the process, we eliminated the work process data of these salespeople. We finally obtained the base observation data of 61 sales personnel. In addition, we have also obtained the quantity information of product sales objects in various regions of Wuhan from Baidu Map (https://map.baidu.com/) and Dianping.com (https://www.dianping.com/) to measure the market potential of each region for which each salesperson was responsible. Finally, we calculated the corresponding indicators from the data using the relevant salesperson performance evaluation model.

### Variables and definition

#### Basic indicators of performance evaluation

Enterprise performance appraisal should not only focus on financial figures but need to include indicators such as finance, process and sustainable development to stimulate sales personnel's enthusiasm to the greatest extent (Churchill et al., 1985). According to existing studies on performance evaluation indicators, this paper has selected indicators that reflect the degree of work effort, work attitude, regional market environment, and sales results, considering the availability of data, as follows data, as shown in Table 1.

**Table 1:** The variables and definitions.

**Indicator name**	**Indicator meaning**	**Calculation method**	**Data source**
Working hours (in hours)	The working length reflects how hardworking are the salesperson (Sujan, 1986).	Working hours = End of work time – Start of work time	The data is obtained from the salesperson punching in the sales work data recording system.
The number of customer visits (in units of one)	The number of customer visits indicate the salesperson's work attitude (Lussier and Hall, 2018).	The number of customer visits is measured by adding up the number of times a salesperson visits a customer in a month. Customers can be visited multiple times, and the number of customer visits increases by 1 for each customer visit by a salesperson.	The sales work data recording system can automatically obtain the visit time and positioning when salesperson visits a customer. So we obtain the frequency of customer visits through the records.
Regional market potential (in units of one)	This variable reflects the market consumption potential (Boles et al., 1995).	Regional market potential is measured by the total number of developed and undeveloped customers in the region.	The quantity information of product sales objects in various regions of Wuhan is obtained from Baidu Map and Dianping.com.
Sales (in Yuan)	Monthly sales reflect sales results (Churchill et al., 1985; Ahearne and Lam, 2012).	Sales is measured by adding up the amount of all orders filled by salespeople in a month.	The data is obtained from the sales work data recording system. After a customer places an order, the system automatically generates the order and displays specific sales information, including product name, product unit price, product quantity, total amount, etc.
Order volume (in units of number)	The monthly order volume reflects sales results and selling characteristics (Boles et al., 1999; Morgan and Slotegraaf, 2012).	Order volume is measured by the sum of all orders filled by salespeople in a month.	The data is obtained from the sales work data recording system.
Sales volume (in units of number)	The number of products sold by the salesperson reflects sales results and selling characteristics (Boles et al., 1999).	Sales volume is measured by adding up the number of all items in all orders sold by a salesperson in a month.	The data is obtained from the sales work data recording system.

#### Selection of input and output variables

In work efficiency evaluation, the variables “working hours”, “number of customer visits,” and “regional market potential” are used as the input variables of the DEA model, whereas the variables “order volume” and “sales volume” are used as the output variables. And in salespeople performance evaluation, we introduce a decision tree model to combine work efficiency and work effectiveness, which is the value of sales (Ahearne and Lam, 2012).

## Results

### Estimated integrated DEA model based on Gini impurity to evaluate work efficiency

We select the basic DEA model for calculating the work efficiency of sales staff from three perspectives. Firstly, we selected the CCR model, the input-oriented BCC model and the output-oriented BCC model from the perspective of constant returns to scale and variable returns to scale. Secondly, since previous DEA models are all radial, non-radial DEA models should also be considered. So we chose the non-radial SBM model. Thirdly, in addition to the conventional DEA model based on self-evaluation, the cross-efficiency model based on peer-evaluation should also be considered. We finally chose the CCR model, the input- and output-oriented BCC model, the SBM_C model, the SBM_V model and the cross-efficiency model. And the measured efficiency results were recorded as *E*^*CCR*^, *E*^*I*−*BCC*^, *E*^*O*−*BCC*^, *E*^*SBM*−*C*^, *E*^*SBM*−*V*^, *E*^*CE*^. Based on the self-evaluation and peer-evaluation DEA model, we established a Gini impurity-based integrated DEA model and measured the work efficiency of the sales staff (Peng et al., 2004), denoted as E. The results are shown in Table 2. In order to protect the information of the salespeople, their names are indicated by numbers.

**Table 2:** Comprehensive work efficiency measured by the Gini impurity-based integrated DEA model.

**Name**	**E^I−BCC^**	**E^O−BCC^**	**E^CCR^**	**E^SBM−C^**	**E^SBM−V^**	**E^CE^**	**E**
1	0.51	0.19	0.18	0.13	0.13	0.12	0.21
2	0.70	0.35	0.33	0.24	0.25	0.22	0.34
3	0.42	0.40	0.33	0.22	0.22	0.21	0.30
4	1.00	1.00	0.81	0.57	1.00	0.52	0.81
5	0.63	0.56	0.55	0.39	0.45	0.21	0.46
6	1.00	1.00	1.00	1.00	1.00	0.61	0.93
7	1.00	1.00	1.00	1.00	1.00	0.41	0.90
8	0.37	0.24	0.19	0.14	0.14	0.13	0.20
9	0.54	0.32	0.32	0.29	0.29	0.22	0.33
10	0.57	0.26	0.26	0.21	0.21	0.18	0.28
11	0.56	0.33	0.33	0.26	0.27	0.22	0.33
12	1.00	0.72	0.71	0.40	0.42	0.17	0.56
13	1.00	1.00	0.84	0.70	1.00	0.53	0.84
14	1.00	1.00	1.00	1.00	1.00	0.24	0.87
15	0.39	0.26	0.25	0.18	0.20	0.08	0.22
16	0.99	0.98	0.97	0.91	0.98	0.61	0.91
17	1.00	1.00	1.00	1.00	1.00	0.18	0.86
18	0.53	0.24	0.23	0.19	0.20	0.15	0.25
19	0.82	0.78	0.75	0.65	0.65	0.43	0.68
20	1.00	1.00	1.00	1.00	1.00	0.64	0.94
21	0.40	0.35	0.27	0.22	0.22	0.19	0.27
22	0.94	0.84	0.82	0.65	0.65	0.44	0.72
23	0.67	0.17	0.14	0.11	0.12	0.09	0.21
24	0.74	0.84	0.74	0.50	0.56	0.45	0.63
25	1.00	1.00	0.97	0.57	1.00	0.49	0.84
26	0.84	0.90	0.82	0.52	0.72	0.51	0.72
27	1.00	1.00	1.00	1.00	1.00	0.27	0.88
28	0.75	0.65	0.60	0.43	0.43	0.27	0.52
29	0.92	0.45	0.33	0.26	0.30	0.21	0.41
30	0.94	0.53	0.53	0.29	0.39	0.20	0.48
31	0.55	0.14	0.14	0.12	0.12	0.10	0.19
32	1.00	1.00	1.00	1.00	1.00	0.30	0.88
33	1.00	1.00	1.00	1.00	1.00	0.56	0.93
34	0.28	0.36	0.17	0.13	0.13	0.10	0.19
35	0.39	0.24	0.21	0.16	0.16	0.14	0.21
36	1.00	1.00	0.92	0.60	1.00	0.57	0.85
37	0.57	0.19	0.17	0.14	0.14	0.12	0.22
38	1.00	0.84	0.79	0.62	0.68	0.21	0.69
39	0.99	0.99	0.98	0.90	0.91	0.59	0.89
40	0.88	0.93	0.87	0.80	0.83	0.54	0.81
41	0.34	0.25	0.19	0.11	0.11	0.11	0.18
42	1.00	1.00	1.00	1.00	1.00	0.64	0.94
43	0.74	0.95	0.72	0.25	0.26	0.18	0.51
44	0.83	0.79	0.79	0.64	0.67	0.46	0.69
45	0.72	0.84	0.71	0.48	0.65	0.45	0.64
46	1.00	1.00	1.00	1.00	1.00	0.28	0.88
47	1.00	1.00	1.00	1.00	1.00	0.45	0.91
48	1.00	1.00	1.00	1.00	1.00	0.78	0.96
49	0.38	0.25	0.20	0.15	0.15	0.14	0.21
50	1.00	1.00	1.00	1.00	1.00	0.71	0.95
51	1.00	1.00	1.00	1.00	1.00	0.71	0.95
52	1.00	0.93	0.89	0.54	0.71	0.24	0.71
53	1.00	1.00	1.00	1.00	1.00	0.64	0.94
54	1.00	1.00	1.00	1.00	1.00	0.20	0.86
55	1.00	1.00	0.81	0.59	1.00	0.50	0.81
56	1.00	1.00	1.00	1.00	1.00	0.60	0.93
57	0.80	0.88	0.78	0.50	0.70	0.48	0.69
58	0.86	0.81	0.80	0.61	0.62	0.53	0.70
59	0.98	0.99	0.81	0.51	0.83	0.51	0.77
60	1.00	0.90	0.83	0.61	0.61	0.44	0.73
61	0.64	0.72	0.60	0.35	0.48	0.38	0.52

In order to protect the information of the salespeople, their names are indicated by numbers.

Through the comparative analysis of the efficiency value results in Table 2, some findings should be noted. Firstly, from the perspective of the discriminative power, the results of the integrated DEA model based on Gini impurity have better discriminative power than those of the conventional DEA model. And its efficiency ranking is more discriminative. We can find that the efficiency value measured by this model is similar to that measured by the traditional DEA model in general, which can reflect the overall ranking trend of salespeople. Also, the integrated method has stronger discriminative power for salespeople with similar efficiency values, especially for those who are identified effective by the traditional DEA model. Compared with the conventional DEA model, the discriminative power of the integrated model is enhanced. Moreover, the Gini impurity of the input-oriented BCC model, output-oriented BCC model, CCR model, SBM model with constant returns to scale, SBM model with variable returns to scale, cross-efficiency model and integrated DEA model are 0.76, 0.81, 0.842, 0.84, 0.8, 0.83, and 0.842, respectively. It can be found that the integrated DEA model has the highest Gini impurity. Therefore, combined with the properties of 2.2, it can be argued that the integrated DEA model based on the Gini impurity has a higher discriminative power than the traditional models.

Secondly, the integrated DEA model based on Gini impurity weighting improves the objectivity of the DEA results. And the obtained efficiency values are milder than those from single-type DEA models. For example, the efficiency value of the salesperson with number 4 is 1 when measured by the input-oriented BCC model, the output-oriented BCC model and the SBM model with variable returns to scale, which means the subject's work is efficient. However, when it is measured by other models, the results are varied, indicating the subject's work efficiency is not high, e.g., when measured by the SBM model with constant returns to scale, the efficiency value is 0.57. It can be seen that the choices of different models will lead to varied performance evaluation results, meaning that a single-type DEA model is unstable and relatively aggressive. The integrated DEA with Gini impurity weighting effectively takes advantage of multiple DEA models and improves the discriminative power and objectivity, thus is more suitable for risk-averse decision makers.

### Estimated salesperson performance

Based on the evaluation of work efficiency, we further assessed the sales performance of the company's sales staff, and the results are shown in Table 3.

**Table 3:** Salesperson performance classification based on integrated DEA with Gini impurity.

**Name**	**Work efficiency E**	**Sales**	**Category**
1	0.21	9,240.48	Problematic
2	0.34	16,768.00	Problematic
3	0.3	21,187.48	Bottleneck
4	0.81	62,024.83	Training-needed
5	0.46	18,216.00	Opportunistic
6	0.93	93,073.00	Benchmark
7	0.9	39,802.00	Opportunistic
8	0.2	18,968.84	Bottleneck
9	0.33	18,157.43	Bottleneck
10	0.28	13,278.00	Problematic
11	0.33	16,987.52	Problematic
12	0.56	12,616.00	Strength-sparing
13	0.84	51,546.00	Training-needed
14	0.87	15,610.99	Strength-sparing
15	0.22	11,624.00	Problematic
16	0.91	52,712.00	Less-motivated
17	0.86	20,773.00	Opportunistic
18	0.25	9,346.00	Problematic
19	0.68	73,604.75	Diligent
20	0.94	102,107.04	Benchmark
21	0.27	24,992.04	Bottleneck
22	0.72	68,749.91	Training-needed
23	0.21	3,912.00	Problematic
24	0.63	63,701.98	Training-needed
25	0.84	32,509.00	Opportunistic
26	0.72	76,838.29	Diligent
27	0.88	30,074.00	Opportunistic
28	0.52	59,586.00	Training-needed
29	0.41	19,633.86	Bottleneck
30	0.48	18,452.00	Opportunistic
31	0.19	9,644.22	Problematic
32	0.88	20,882.00	Opportunistic
33	0.93	27,609.00	Opportunistic
34	0.19	28,826.30	Bottleneck
35	0.21	14,200.00	Problematic
36	0.85	89,316.80	Benchmark
37	0.22	11,362.00	Problematic
38	0.69	19,798.00	Opportunistic
39	0.89	89,563.38	Benchmark
40	0.81	78,951.18	Benchmark
41	0.18	15,170.20	Problematic
42	0.94	59,569.00	Less-motivated
43	0.51	90,834.00	Diligent
44	0.69	75,650.00	Diligent
45	0.64	82,602.84	Diligent
46	0.88	19,690.00	Opportunistic
47	0.91	84,910.00	Benchmark
48	0.96	66,727.75	Less-motivated
49	0.21	17,628.00	Bottleneck
50	0.95	57,887.48	Less-motivated
51	0.95	59,424.69	Less-motivated
52	0.71	14,886.00	Strength-sparing
53	0.94	57,753.44	Less-motivated
54	0.86	4,094.00	Strength-sparing
55	0.81	81,505.02	Diligent
56	0.93	70,088.32	Less-motivated
57	0.69	80,961.10	Diligent
58	0.7	49,189.33	Training-needed
59	0.77	88,071.88	Diligent
60	0.73	58,974.16	Training-needed
61	0.52	59,988.59	Training-needed

In order to protect the information of the salespeople, their names are indicated by numbers.

**Figure 3:**
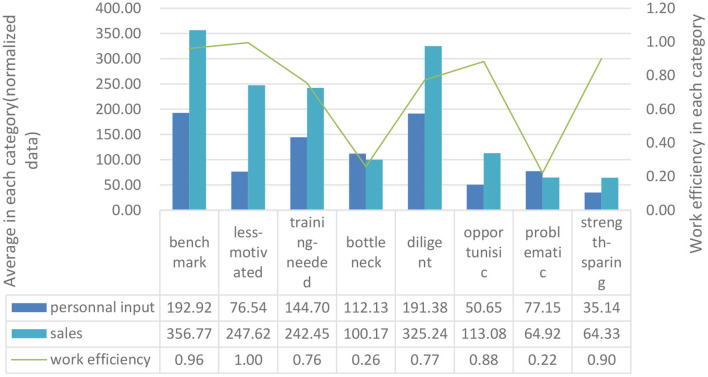
Average personnel input, work efficiency and sales in each type of salesperson.

From Table 3, the following performance diagnoses can be drawn. First of all, compared with the performance evaluation model examining only work output, this model establishes an effective connection between salespeople's work process and work effect. And it can comprehensively and effectively make more accurate evaluations for all kinds of salespeople. The average work input, average work efficiency and average sales for each type of salesperson are summarized in Figure 3, which validates the accuracy of the salesperson performance evaluation model's characteristics for each type of salesperson. Figure 3 indicates that it is unreasonable to use work efficiency or sales alone as the performance evaluation criterion. For example, the work efficiency of the salesperson with number 61 is 0.52, 17.1% lower than the overall average and 19.3% lower than the group average. However, his sales are $59,988.59, 37.5% higher than the total average and 33.1% higher than the group average. The performance appraisal model classifies ^*^Lei as the training needed-type salesperson, having the characteristics of medium input, medium output and comparatively low work efficiency. In other words, the input and output of this type of salesperson are disproportionate, and they have relatively low work efficiency. Therefore, companies need to enhance their skills through training to improve their work performance. Another example is the salesperson with number 56, whose work efficiency is 0.93, 48.4% higher than the total average and 44.3% of the group average. Her sales are $70,088.32, 60.6% higher than the total average and 55.5% higher than the group average. The performance appraisal model identified her as a less-motivated type, indicating that she needs to be motivated to increase work input. Combined with the work input of less-motivated salespeople, this type of employee has the characteristics of medium input, comparatively high output and high work efficiency. The average working time is 57.04, which is lower than 36.3% of the total average, and the average number of customer visits is 11.56, which is lower than 41.7% of the total average. Therefore, for less-motivated salespeople, companies should increase their work engagement by motivating employees to improve performance. Similarly, some salespeople are judged to be benchmarks, that is, such employees have the benchmarking characteristics of high input-high output-high work efficiency.

Secondly, the model effectively quantifies the impact of work efficiency on sales results. For instance, the diligent-type salespeople exceed the performance of benchmark-type salespeople in categories like the working hours, the number of customers visits and the regional market potential by 6.7, 2, and 1.6%, but their performance in sales was significantly lower than the benchmark-type salespeople. The research found that the diligent-type salespeople are 22.5% lower than the benchmark type in work efficiency, which could be a reasonable explanation. Therefore, the model quantifies the impact of work efficiency on sales results, and can then provide targeted promotion strategies for different types of salespeople.

Finally, the performance evaluation model effectively considers the impact of market resources on an individual's sales performance. The research has found there is no significant correlation between salesperson performance and market resource allocation. The results show that the factor of the regional market potential in the regions where the benchmark, diligent, less-motivated and training-needed salespeople are located is smaller than that of the other groups, but the sales are all higher than the other groups, which shows that compared with the external market potential, salespeople's work process is key to their sales performance.

## Discussion and conclusion

Drawing on multiple DEA models, this study has proposed an integrated DEA model by integrating self- and peer-evaluation DEA models and Gini impurity. And taking the sales data of a Chinese liquor company as the case, the study has also incorporated indicators such as working hours, customer visits, and regional market potential into the DEA model to obtain the sales staff's work efficiency. The results show that the new model is more stable and objective than traditional DEA models, and it has stronger discriminative power on salespeople with similar efficiency values. Furthermore, in order to comprehensively and effectively assess the performance of sales personnel and balance the work process and results in the salespeople performance evaluation system, we have constructed a new performance evaluation system based on the decision tree model, which considers both sales results and process performance. Using the new evaluation system, we measured the sales staff's process performance by work efficiency and their sales results by sales volume. Based on the model results, we have divided salespeople into eight types: benchmark, diligent, less-motivated, training-needed, opportunistic, bottleneck, strength-sparing and problematic salespeople. We have found that the benchmark and diligent salespeople have much higher work input than other types, and achieve better sales results. In addition, the work efficiency of less-motivated, opportunistic and strength-sparing salespeople is much higher than other types of salespeople having the same sales results. But because of their insufficient work effort, they do not achieve high sales volume matching their high work efficiency. The training-type and bottleneck-type salespersons have insufficient work ability, so their sales results are lower than expected due to low work efficiency when the same work input is performed. Problematic-type salespeople are the most ineffective among the eight types, and their poor performance should be ascribed to their poor work ability.

Based on the above findings, we have proposed the following suggestions for optimizing performance management. First, the company should train the diligent, training-needed and bottleneck salespeople. These salespeople's biggest problem is lower work efficiency, leading to their lower sales performance. Companies can train these salespeople to improve their efficiency and thus performance. Second, companies should encourage less-motivated, opportunistic and strength-sparing salespeople to increase their work engagement. Although these salespeople have high work efficiency, they do not achieve high sales performance because of their limited work engagement. Therefore, promoting their work investment will significantly improve sales performance. Third, for the problematic-type salespeople, since their work ability and input are seriously insufficient, they must participate in training and increase work investment to enhance performance.

This study also suffers from several limitations that should be taken into account when interpreting its results. We examined the effectiveness of the performance evaluation model using only liquor salespeople in the B2B industry as an example; however, like many studies in this domain, we collected the data from one industry and the model of the research needs to be optimized to fit other industry contexts. Although our research serves this purpose, further research is needed to better understand salespeople performance evaluation method and to test our model further across industries and business contexts.

## Data availability statement

The original contributions presented in the study are included in the article/supplementary material, further inquiries can be directed to the corresponding authors.

## Author contributions

AC: methodology, investigation, formal analysis, writing—original draft, visualization, and writing—review and editing. ML: conceptualization, data curation, and funding acquisition. HL: methodology, validation, and writing—review and editing. All authors contributed to the article and approved the submitted version.

## Funding

This research was supported by Humanities and Social Science Planning Project of Ministry of Education (No. 18YJA63006).

## Conflict of interest

The authors declare that the research was conducted in the absence of any commercial or financial relationships that could be construed as a potential conflict of interest.

## Publisher's note

All claims expressed in this article are solely those of the authors and do not necessarily represent those of their affiliated organizations, or those of the publisher, the editors and the reviewers. Any product that may be evaluated in this article, or claim that may be made by its manufacturer, is not guaranteed or endorsed by the publisher.
